# Case Report: FIA plus venetoclax in a patient on hemodialysis

**DOI:** 10.3389/fonc.2025.1740357

**Published:** 2026-01-15

**Authors:** Jacob E. Herstein, Kayleigh R. Marx, J. Michael Savoy, Wei Ying Jen, Musa Yilmaz, Nicholas J. Short, Ghayas C. Issa, Farhad Ravandi, Tapan M. Kadia, Naval G. Daver, Courtney D. DiNardo

**Affiliations:** 1 Department of Internal Medicine, University of Texas Health Science Center at Houston, Houston, TX, United States; 2 Division of Pharmacy, University of Texas MD Anderson Cancer Center, Houston, TX, United States; 3 Department of Leukemia, University of Texas MD Anderson Cancer Center, Houston, TX, United States

**Keywords:** acute myeloid leukemia, case report, chemotherapy, hematologic oncology, hemodialysis

## Abstract

The safe and effective delivery of curative cytotoxic chemotherapy for acute myeloid leukemia (AML) in patients receiving intermittent hemodialysis (IHD) for end-stage renal disease (ESRD) remains a clinical challenge; pharmacological and logistical barriers necessitate close interdisciplinary coordination. In this case, we report a 65-year-old female patient with chronic ESRD on IHD and newly diagnosed AML who achieved complete remission (CR) after treatment with fludarabine, idarubicin, and cytarabine plus venetoclax (i.e., FIA + venetoclax).

## Introduction

Optimal strategies for the safe administration of chemotherapy in patients with chronic kidney disease (CKD) are not well-established, in part due to the exclusion of such patients from most clinical trials (1). At the present time, most recommendations lack strong clinical support and are based predominantly on consensus opinions rather than evidence-based guidelines. Despite attempts to standardize dose adjustments and chemotherapy timing, the absence of substantial pharmacokinetic data along with the development of novel, multi-agent chemotherapy regimens is a persistent obstacle (2, 3). This case report outlines an approach for the administration of fludarabine, idarubicin, and cytarabine (FIA) plus venetoclax in patients receiving intermittent hemodialysis (IHD) for end-stage renal disease (ESRD).

## Case

The patient was a 65-year-old woman with hypertension, hyperlipidemia, multi-vessel coronary artery disease, insulin-dependent type 2 diabetes mellitus, renal cell carcinoma status-post left partial nephrectomy, and ESRD requiring IHD three times weekly with associated anemia and mineral bone disorder. She initially sought medical attention due to complaints of fatigue, dyspnea, generalized weakness, and malaise, as well as increasing transfusion requirements for chronic anemia attributed to CKD. Laboratory results on presentation were significant for white blood cell count (WBC) 41.8 × 10^9^/L (absolute neutrophil count 15.59 × 10^9^/L and absolute monocyte count 17.26 × 10^9^/L), hemoglobin 6.4 g/dL, and platelet count 144 × 10^9^/L. Bone marrow biopsy showed 24% blasts and confirmed acute myeloid leukemia (AML) with myelomonocytic differentiation. Bone marrow flow cytometry was notable for 25.3% blasts (CD45 dim with intermediate side scatter) with myelomonocytic differentiation, including a population of myeloblasts (7% of total events) and a population of aberrant monocytic precursors (18% of total events). Next-generation sequencing (NGS) revealed mutations in *DNMT3A* and *PTPN11*. *FLT3* analysis was negative for internal tandem duplication (ITD) and tyrosine kinase domain (TKD) mutations. The karyotype was 46,XX[20]; optical genome mapping and RNA gene fusion panel demonstrated intragenic recombination of the *KMT2A* gene, suggestive of partial tandem duplication of *KMT2A*.

The patient was also found at presentation to have a large malignant pericardial effusion requiring pericardial drain and subsequent window. She received hydroxyurea 2 g daily for 1 week with adequate cytoreduction to WBC 6.2, and after a multidisciplinary conference weighing risks and benefits of treatment approaches, she was subsequently initiated on renally dosed and attenuated intensive chemotherapy with 3-day FIA plus venetoclax with additional dose reductions due to performance status (Eastern Cooperative Oncology Group Performance Status of 2) and age >65 (**Table 1**). The patient was transferred out of the intensive care unit but remained hospitalized to allow for the coordination of daily IHD and tumor lysis syndrome (TLS) monitoring. Chemotherapy was scheduled for early morning administration to allow for scheduled IHD approximately 6 hours after the completion of the cytarabine infusion. Volumes of ultrafiltration varied according to patient volume status and laboratory results, ranging from 1,200 to 2,000 mL. An additional IHD session was scheduled in the morning of day 4, following the conclusion of FIA, to adjust the patient back to the previous baseline dialysis cadence. She met laboratory diagnostic criteria for TLS (4) on cycle 1, day 1 (C1D1), but no specific intervention was required, given that she was already scheduled to undergo hemodialysis. She did not experience significant cardiac or neurological toxicities. She had full peripheral blood count recovery by day 26, and a bone marrow biopsy on day 28 confirmed complete remission (CR1) with no evidence of measurable residual disease (MRD) by multiparameter flow cytometry (assay validated to a sensitivity of 0.1%). She has since completed an additional three cycles of consolidation therapy using FIA plus venetoclax with ongoing remission. Anthracycline use was omitted for consolidation courses; fludarabine and cytarabine dosing was maintained, given a positive treatment response and acceptable toxicity of induction cycles.

**Table 1:** Detailed dosing and administration of FIA+VEN regimen with intermittent hemodialysis. Adjustments made according to renal function, performance status, age, and concomitant medications.

Chemotherapy	Induction dosing and administration^€^	Consolidation cycles 2 and 4^¥^
Fludarabine	15 mg/m^2^ IV on days 1–3, starting daily at 0530	15 mg/m^2^ IV on days 1–3, starting daily at 1900
Idarubicin	6 mg/m^2^ IV on days 1 and 2, administered at 0700	N/A
Cytarabine	400 mg/m^2^ on days 1–3; infused over 3 hours, starting 3 hours after completion of fludarabine at 0900	400 mg/m^2^ on days 1–3; infused over 3 hours, starting 3 hours after completion of fludarabine at 2200
Venetoclax	50 mg PO on day 2, followed by 100 mg PO on day 3 and 200 mg PO on days 4–6; target dose 400 mg reduced for concomitant amiodarone	100 mg daily on days 1–3; target dose 400 mg reduced due to concomitant voriconazole
Hemodialysis	Daily C1D1–C1D4 starting 6 hours after completion of cytarabine infusion at 1800; duration and volume of ultrafiltration tailored to volume status and laboratory studies	Daily D2–4 starting at 0800

^€^ Timing approximated to nearest half hour.

^¥^ C3 consolidation chemotherapy timing and intermittent hemodialysis (IHD) administration as per induction.

## Discussion

Review of existing literature confirmed a paucity of evidence for treating AML in patients already requiring IHD at the time of diagnosis; previously published reports are largely limited to either more traditional induction regimens or hypomethylating agents in combination with venetoclax for those who are not candidates to undergo intensive induction (5–7). This report provides an alternative dosing strategy when either continuous cytarabine is not feasible or higher peak concentrations are more desirable. The use of fludarabine-containing regimens, utilizing synergistic sequencing that optimizes the accumulation of active Ara-C triphosphate within AML blasts, may provide additional therapeutic advantage in patients requiring renally dose-adjusted regimens for toxicity mitigation (8). In patients with ESRD on IHD receiving FIA plus venetoclax for newly diagnosed or relapsed or refractory (R/R) AML, we offer the following considerations and recommendations:

- Approximately 60% of fludarabine’s active metabolite, free nucleoside 9-beta-d-arabinosyl-2-fluoroadenine (F-Ara-A), is renally excreted (9). Fludarabine dosing should be reduced to 50% of the planned dose and given 3 hours prior to cytarabine (10).
- Cytarabine should be reduced by at least 50%. Assuming a full FIA dose of 2,000 mg/m^2^, we advocate reducing to at least 1,000 mg/m^2^ total, although a subsequent reduction to 500 mg/m^2^ is reasonable if the patient has poor performance status or age >65 years. Close coordination with nephrology is necessary, given the importance of daily hemodialysis 2–6 hours after the end of the cytarabine infusion to minimize toxicity; this is particularly important for limiting the risk of neurotoxicity associated with the uracil arabinoside (Ara-U) metabolite, which is known to have higher area under the curve (AUC) and longer half-life in patients with underlying renal dysfunction (11–13). Notably, Ara-U is effectively dialyzable, and several case reports have previously demonstrated the efficacy of IHD in reducing the likelihood of neurotoxicity (14–16). Infusion rate should be slowed to deliver the drug over at least 3 hours to avoid high peak serum concentrations. In accordance with established guidelines, we recommend a thorough neurological assessment prior to each dose of cytarabine with particular attention to signs of cerebellar dysfunction; administration on an inpatient unit with oncology-trained nursing staff utilizing standardized neurological assessment tools is recommended (17, 18).
- While it is well-established to consider a 50% dose reduction for daunorubicin in patients with creatinine > 3 mg/dL, the optimal approach for idarubicin is less clear (10, 19). Some consider reducing the dose of idarubicin by 30%; however, doses used in FIA are lower than those in the classic “7 + 3” regimen (20, 21). For AML with an *FLT3* mutation receiving a targeted FLT3 inhibitor, we would similarly recommend idarubicin 6–8 mg/m^2^ with close monitoring. These doses are approximately 35% lower than the traditional 7 + 3.
- Venetoclax should be initiated using a 3–4-day ramp-up dosing schedule as per manufacturer recommendations to reduce the risk of TLS. While no adjustment is required for renal impairment, patients may require closer monitoring for TLS and more aggressive prophylaxis. It is hepatically metabolized and should be dose-reduced in patients receiving moderate or strong CYP3A inhibitors or P-gp inhibitors.
- In patients already receiving thrice-weekly dialysis, it is preferable to increase the frequency to daily dialysis during the administration of chemotherapy.
- Timing of chemotherapy and hemodialysis should be adjusted in a manner congruent with institutional workflows. The patient described in our case has received four total courses of chemotherapy with daily IHD support. Cycle 1, as described, utilized a morning administration of chemotherapy and a late afternoon IHD schedule. During consolidation, alternative scheduling with late afternoon chemotherapy administration followed by morning IHD was leveraged and may be considered based on institutional chemotherapy cutoff times and dialysis provider availability. There were no adverse events or notable differences in toxicity associated with scheduling changes.

With careful dose adjustments and care coordination, we maintain that curative intent therapy using the FIA + VEN regimen remains an achievable goal for patients with ESRD or acute kidney injury requiring IHD at the time of AML diagnosis.

## Data Availability

The original contributions presented in the study are included in the article/supplementary material. Further inquiries can be directed to the corresponding author.
